# Laser-assisted delivery enhances topical uptake of the anticancer agent cisplatin

**DOI:** 10.1080/10717544.2018.1534896

**Published:** 2018-11-26

**Authors:** Emily Wenande, Uffe H. Olesen, Malene R. Boesen, Daniel P. Persson, Catharina M. Lerche, Stefan Stürup, Bente Gammelgaard, Søren Husted, R. Rox Anderson, Merete Haedersdal

**Affiliations:** a Department of Dermatology, Bispebjerg Hospital, University of Copenhagen, CopenhagenNV, Denmark;; b Wellman Center for Photomedicine, Massachusetts General Hospital, Harvard Medical School, Boston, Massachusetts, USA;; c Department of Pharmacy, Faculty of Health and Medical Sciences, University of Copenhagen, Copenhagen Ø, Denmark;; d Department of Plant and Environmental Sciences, Faculty of Science, University of Copenhagen, Frederiksberg C, Denmark

**Keywords:** Element bioimaging, fractional ablative CO_2_ laser, laser-assisted drug delivery, local chemotherapy, non-melanoma skin cancer

## Abstract

Systemic chemotherapy with the anticancer agent cisplatin is approved for advanced non-melanoma skin cancer (NMSC), but topical treatment is limited by insufficient cutaneous penetration. We studied the impact of ablative fractional laser (AFL) exposure on topical cisplatin’s pharmacokinetics and biodistribution in skin, using microscopic ablation zones reaching the mid- (MAZ-MD; 620 μm depth) and deep dermis (MAZ-DD; 912 μm depth) (*λ* = 10,600 nm, 196 MAZ/cm^2^). Assessed in an *in vitro* Franz cell model after 0.5-, 4-, 24 h topical exposure (*n* = 8), cisplatin delivery was greatly accelerated by AFL, shown by quantitative- and imaging-based inductively coupled plasma-mass spectrometry (ICP-MS). After 30 minutes, cisplatin concentrations were 91.5, 90.8 and 37.8 μg/cm^3^ in specific 100-, 500, and 1500 μm skin layers respectively, contrasting to 8.08, 3.12, 0.64 μg/cm^3^ in non-laser-exposed control skin (*p* < .001; control vs MAZ-MD). Supported by element bioimaging, the greatest relative increases occurred in the deep skin compartment and at later time points. After 24 h, cisplatin concentrations thus rose to 1829, 1732 and 773 μg/cm^3^, representing a 25-, 103- and 447-fold enhancement in the 100, 500, and 1500 μm deep skin layers versus corresponding controls (*p* < .001; MAZ-MD). A significant difference in cutaneous uptake using MAZ-MD and MAZ-DD was not shown at any time point, though deeper laser channels resulted in increased transdermal cisplatin permeation (*p* ≤ .015). In conclusion, AFL is a rapid, practical and existing skin treatment that may provide greatly enhanced uptake of topical cisplatin for treatment of superficial and deep skin cancer.

## Introduction

Since its approval in 1978, cisplatin (300.1 g/mol) has remained one of the most successful and versatile anticancer agents in oncology. Used as both mono- and combination therapy, the drug represents a cornerstone treatment of epithelial cancers of the head and neck, lungs, testes, ovaries and bladder (Chabner et al., 2015). On the molecular level, cisplatin is first activated by hydrolysis inside the cell, subsequently forming intra- and interstrand crosslinks with DNA. The resulting distortion in DNA structure leads to G2/M cell cycle arrest, inhibition of replication, transcription and ultimately, cellular apoptosis (Chabner et al., 2015).

The activity of cisplatin against non-melanoma skin cancer (NMSC) is established in both preclinical and clinical settings. Cisplatin causes lethality in multiple NMSC cell lines (Featherstone et al., 1991, Gil et al., 2016, Olesen et al., 2017), and systemic cisplatin-based chemotherapy is conventionally used for both locally-advanced and metastatic NMSC (Moeholt et al., 1996, Bejar & Maubec, 2014, Trodello et al., 2017). The agent’s usefulness is however offset by notable toxicity following intravenous administration (Chabner et al., 2015, Chang et al., 2018). An effective treatment using topical cisplatin would be far less toxic, but is limited by insufficient penetration into the skin likely due to the agents’ poor lipophilicity (reported range of experimental Log*P*: -2.55 − -2.19) (Leo et al., 1995, Ghezzi et al., 2004).

Local delivery strategies have the potential to maximize target effects while sparing off-target tissue (Marwah et al., 2016). Efforts to develop topical cisplatin-based therapies have prompted the application of multiple enhancement techniques, including chemo-coated microneedles, iontophoresis, intralesional depot injection, electrochemotherapy, cisplatin bead implantation and a range of chemical enhancers (Chang et al., 1993, Kitchell et al., 1995, Sersa et al., 1998, Bacro et al., 2000, Hewes & Sullins, 2006, Simonetti et al., 2009, Gupta et al., 2014, Byrne et al., 2015, Uddin et al., 2015). Laser-assisted drug delivery (LADD) constitutes an alternative strategy that has gained clinical impact as a practical, highly effective, and customizable cutaneous delivery modality. By generating microscopic ablation zones (MAZs) in the skin using an ablative fractional CO_2_ or erbium laser (AFL), the skin’s natural barrier function is compromised. The resulting increase in cutaneous porosity improves bioavailability of topically-applied agents. To date, use of LADD is reported for a range of indications including neoplastic skin lesions, though cisplatin has yet to be studied (Haedersdal et al., 2016, Wenande et al., 2017a).

Recently, we showed that AFL-assisted delivery provides increased uptake of the anticancer agents 5-fluorouracil (5-FU) and methotrexate in *ex vivo* skin, with greater enhancement using deeper laser channels (Taudorf et al., 2015, Wenande et al., 2017b). With the overall perspective to develop a potent, local treatment strategy for NMSC using chemotherapeutic agents, this study assesses the impact of AFL on cutaneous uptake of cisplatin, measuring pharmacokinetics and drug biodistribution using two different laser-channel depths.

## Materials & methods

### Study design

**Figure 1.**
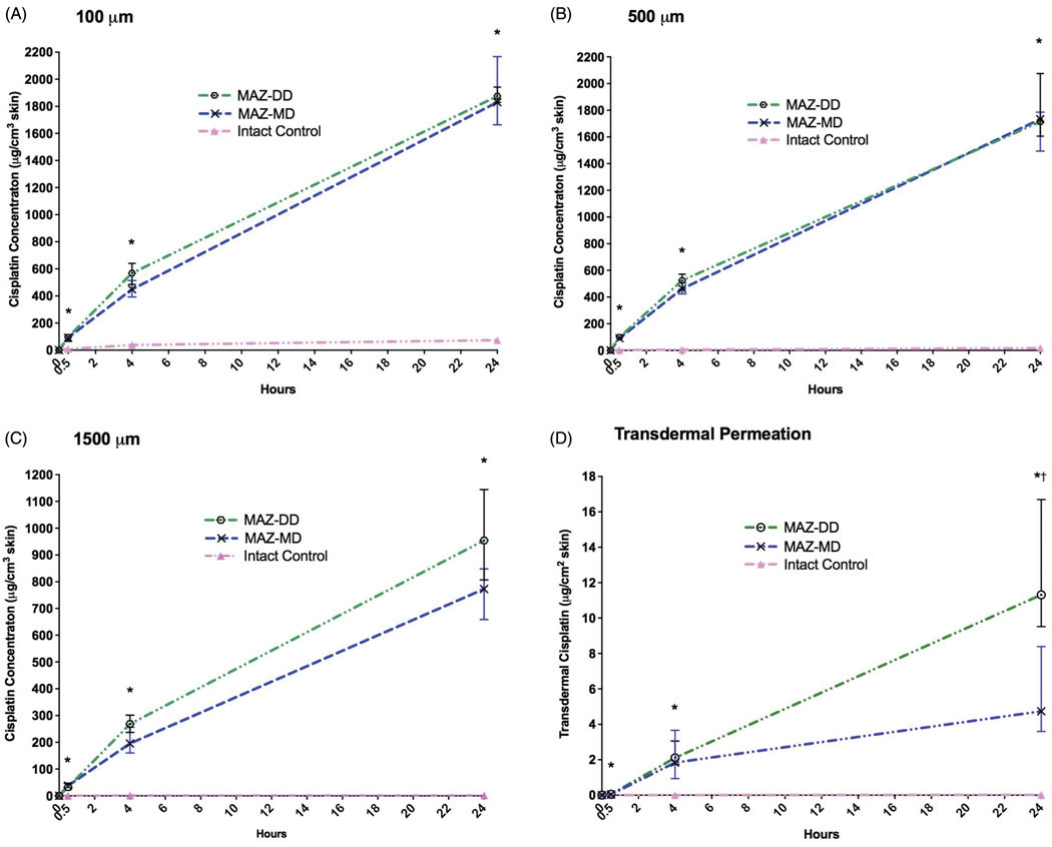
Impact of ablative fractional laser (AFL) delivery on intracutaneous cisplatin biodistribution at skin depths of (A) 100 μm, (B) 500 μm and (C) 1500 μm as well as (D) transdermal cisplatin permeation versus non-laser-exposed controls. Concentrations are presented as medians with interquartile ranges. Compared to control samples, AFL-treated skin with microscopic ablation zones reaching the mid- (MAZ-MD) and deep dermis (MAZ-DD) showed statistically significant (*) enhanced drug uptake in all examined skin layers, culminating at 24 h. In the deepest skin compartment, deposition increased steadily, fitted to power regression models for both laser channel depths (MAZ-DD: cisplatin_1500μm_ (μg/cm^3^) = 65.103 * time_(h)_^0.875^; *R*^2^ = 0.957; MAZ-MD: cisplatin_1500μm_ (μg/cm^3^) = 65.520 * time_(h)_^0.775^; *R*^2^ = 0.973). Increasing laser channel depth did not significantly affect degree of cisplatin delivery in any skin layer. On the other hand, transdermal permeation was found to be significantly greater (†) using deeper channels after 24-h cisplatin diffusion (MAZ-MD vs MAZ-DD, *p* = .001).

**Figure 2.**
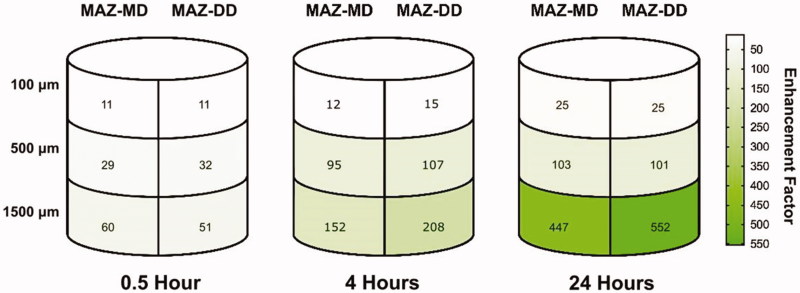
Schematic illustration of relative enhancements in cisplatin deposition within specific skin layers using microscopic ablation zones reaching the mid- and deep dermis compared to non-laser exposed skin (MAZ-MD: 620 μm; MAZ-DD: 912 μm). Overall, impact of laser-assisted cisplatin delivery was most pronounced in deep skin layers and at later time points, resulting in a maximum 552-fold enhancement by 24 h in the 1500 µm skin compartment (control skin vs MAZ-DD, *p* = .001). Cutaneous cisplatin concentrations provided by MAZ-MD and MAZ-DD were not significantly different (*p* ≥ .084), although a tendency to greater deep drug delivery by MAZ-DD was observed at later time points.

**Figure 3.**
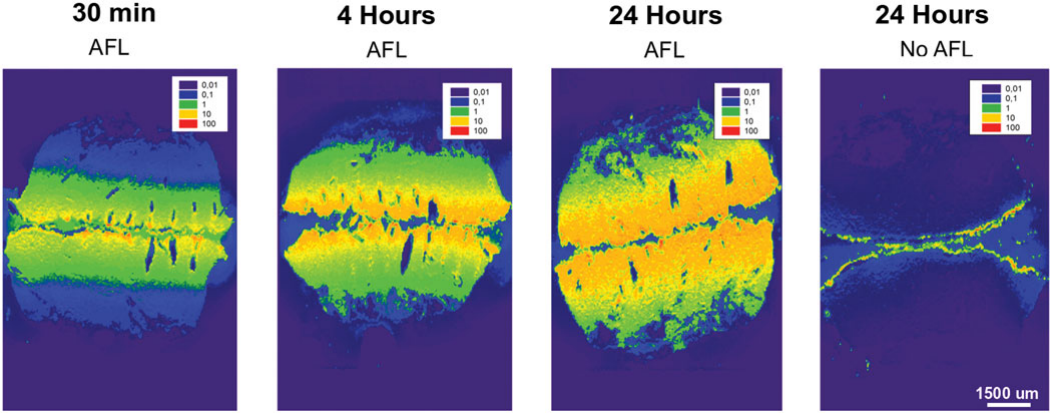
Visualization of cutaneous cisplatin biodistribution with and without laser delivery (MAZ-DD), imaged by laser ablation inductively coupled plasma-mass spectrometry (LA-ICP-MS) in bisected samples (superficial skin layers appear medially in images). Cisplatin biodistribution is presented normalized against endogenous ^13^C. To further show relative skin distribution in individual samples, an internal normalization was used where the highest cisplatin detection points in each sample was set to an index of 100 (see scale bar). Remaining data points were then multiplied with the same correction factor. AFL delivery led to accelerated and enhanced cisplatin deposition within 30 minutes of topical drug application, with progressively greater and deeper uptake over time. Reaching maximal delivery at 24 hours, substantial drug detection was noted extending to the deepest layers of skin AFL samples. In contrast in non-laser-exposed samples, cisplatin remained confined to the outermost skin layers despite topical exposure for 24 h.

Impact of AFL delivery on topical cisplatin diffusion was investigated in a total of 80 fresh frozen, full-thickness porcine skin samples using an *in vitro* Franz cell (FC) model. Intervention groups (*n* = 8) comprised skin exposed to AFL using two different laser-channel depths and non-laser-exposed control skin. After 0.5, 4 or 24 h topical cisplatin exposure, cutaneous drug biodistribution was assessed in three specific skin depths (100, 500 and 1500 μm) using inductively coupled plasma-mass spectrometry (ICP-MS). Measurement of cisplatin content in FC receiver compartments evaluated transdermal cisplatin permeation (Table 1, Figures 1 and 2). Laser ablation ICP-MS (LA-ICP-MS) imaging provided semi-quantitative visualization of cisplatin kinetics and biodistribution in selected MAZ-DD and control samples (Figure 3).

**Table 1.** Study design and ICP-MS quantification of cisplatin delivery in AFL-exposed and intact control skin.

Intervention & time (hours)	*n*	Cryosection depth/sample type	Quantified platinum (median and IQR) (ng/ml)	Calculated cisplatin Concentration† (μg/cm^3^)	Calculated cisplatin Concentration‡ (μg/cm^2^)	*p*-value¥
*Cisplatin + intact skin*						
0.5 h	8	100 µm	6.10 (3.19–7.65)	8.08 (4.22–10.14)	–	–
		500 µm	2.36 (1.75–3.24)	3.12 (2.32–4.29)	–	–
		1500 µm	0.48 (0.29–0.66)	0.64 (0.38–0.87)	–	–
		Receiver	0.35 (0.15–1.39)	–	5.5 × 10^−4^	–
4 h	8	100 µm	29.0 (25.0–48.8)	38.4 (33.2–64.7)	–	–
		500 µm	3.69 (2.15–9.70)	4.88 (2.85–12.8)	–	–
		1500 µm	0.98 (0.38–2.96)	1.29 (0.50–3.93)	–	–
		Receiver	0.42 (0.15–10.6)	–	6.6 × 10^−4^	–
24 h	8	100 µm	55.6 (41.7–63.1)	73.8 (55.3-83.7)	–	–
		500 µm	12.8 (6.63-21.8)	16.9 (8.79–28.9)	–	–
		1500 µm	1.31 (0.66–1.78)	1.73 (0.87–2.36)	–	–
		Receiver	8.22 (1.83–24.6)	–	0.0128	–
*Cisplatin + MAZ-MD*^a^						
0.5 h	8	100 µm	69.0 (64.3–85.3)	91.5 (85.2–113)	–	<.001
		500 µm	68.5 (64.3–89.0)	90.8 (85.2–118)	–	<.001
		1500 µm	28.5 (23.3–35.3)	37.8 (30.8–46.8)	–	<.001
		Receiver	26.0 (19.5–54.0)	–	0.0406	.015
4 h	8	100 µm	338 (296–387)	449 (392–514)	–	.006
		500 µm	349 (320–382)	463 (424–506)	–	.012
		1500 µm	147 (121–193)	195 (160–256)	–	.003
		Receiver	1176 (599–2343)	–	1.838	<.001
24 h	8	100 µm	1380 (1255–1861)	1829 (1664–2468)	–	<.001
		500 µm	1307 (1127–1347)	1732 (1494–1786)	–	<.001
		1500 µm	583 (497–639)	773 (658–878)	–	<.001
		Receiver	3031 (2300–5370)	–	4.735	<.001
*Cisplatin + MAZ-DD*^b^						
0.5 h	8	100 µm	72.0 (49.0–75.3)	95.4 (65.0–99.8)	–	<.001
		500 µm	74.5 (67.3–80.5)	98.7 (89.2–107)	–	<.001
		1500 µm	24.5 (18.5–35.8)	32.5 (24.5–47.4)	–	<.001
		Receiver	31.5 (17.3–49.3)	–	0.0492	.015
4 h	8	100 µm	247 (366–483)	568 (485–641)	–	<.005
		500 µm	396 (345–431)	524 (457–571)	–	<.005
		1500 µm	203 (179–227)	268 (237–301)	–	<.005
		Receiver	1348 (1106–1955)	–	2.107	<.005
24 h	8	100 µm	1414 (1380–1465)	1874 (1829–1942)	–	<.001
		500 µm	1293 (1211–1565)	1713 (1606–2076)	–	<.001
		1500 µm	720 (608–863)	954 (807–1144)	–	<.001
		Receiver	7243 (6092–10687)	–	11.316	<.001
*NaCl + MAZ-DD*						
*(negative control)*	8	100 µm	<1.00	–	–	–
24 h		500 µm	<1.00	–	–	–
		1500 µm	<1.00	–	–	–
		Receiver	<1.00	–	–	–

AFL: ablative fractional laser.†Calculated based on quantified platinum and skin volume (surface area × thickness), where individual skin section volume was 0.2515 cm^2^×0.003 cm.

‡ Calculated based on a known receiver fluid volume and a skin surface area of 0.64 cm^2^.

¥ Adjusted *p*-value comparing cisplatin uptake in AFL-exposed samples and non-laser exposed controls at corresponding time points.

a laser delivery via microscopic ablation zones reaching the mid-dermis (MAZ-MD; 620 µm).

b laser delivery via microscopic ablation zones reaching the deep dermis (MAZ-DD; 912 µm).

### Skin preparation

Immediately after euthanasia, full-thickness skin from the flank of one female Danish Landrace pig (3 months, 34 kg) was surgically excised. Skin was stored for up to twenty days at -80 °C and thawed to room temperature at study initiation. During preparation, excessive hairs and subcutaneous fat were removed using an electric trimmer and scalpel, respectively. Care was taken to avoid nicking or scraping the skin surface.

### Laser irradiation and channel dimensions

AFL was performed using an Ultrapulse CO_2_ laser DeepFx handpiece (λ = 10,600 nm, Lumenis, Santa Clara, CA, USA) at 0.12 mm spot size, 5% density (196 MAZ/cm^2^) and pulse energies of 25 mJ/microbeam (mJ/mb) or 50 mJ/mb. This created an array of MAZs extending to the mid-dermis (MAZ-MD) and deep dermis (MAZ-DD) respectively, determined by blinded measurement of distinct channels in histological cross-sections (*n* = 10). Median MAZ-MD and -DD depths were 620 µm (interquartile range (IQR) 570-689 µm) and 912 µm (840–1063 µm), while corresponding surface ablation widths were 108 µm (98–126 µm) and 130 µm (121–141 µm), respectively. Coagulation zone (CZ) thicknesses along the margins of MAZs, were 46 µm (41–49 µm) and 51 µm (46–54 µm). Total ablated volume of 196 MAZs/cm^2^ was calculated based on the volume of a cone (⅓ × π (½ × width [cm])^2^ × depth [cm] × number of MAZs/cm^2^), resulting in 37.1 × 10^−5 ^cm^3^/cm^2^ skin area for MAZ-MD and 79.8 × 10^−5 ^cm^3^/cm^2^ skin area for MAZ-DD.

### Franz cell diffusion model

Within 30 minutes of laser exposure, full-thickness skin samples were mounted on a FC model (PermeGear Inc., Hellertown, PA, USA) consisting of a donor and receiver compartment. Skin sample stratum corneum (0.64 cm^2^) faced donor compartments, while receiver compartments contained 5.5–5.8 ml phosphate buffered saline (pH 7.4, 37 °C) and a magnetic stir bar. At baseline, 1 ml commercially available IV cisplatin solution (1 mg/mL) (Accord Healthcare Limited, Middlesex, UK) corresponding to 1 mg cisplatin per FC (1.56 mg/cm^2^), was topically applied to skin samples via donor compartments. Prior to sealing FCs with Parafilm® (Bemis, Oshkosh, WI, USA), skin impedance measurement was used to confirm barrier disruption in laser-treated samples and an intact barrier in control skin (mean: 4.70 kΩ (AFL skin); 58.42 kΩ (intact skin)) (Prep-check Electrode Impedance Meter 30 Hz, General Devices, Ridgefield, NJ, USA).

At 0.5, 4 or 24 h diffusion time, skin samples were dismounted, padded dry, and biopsied using an 8-mm punch (0.503 cm^2^ surface area). A biopsy half from each sample was then mounted on Tissue Tek^®^O.C.T™ Compound (Sakura Finetek Europe B.V., Alpehn aan den Rijn, Netherlands), snap frozen, cut in 30 µm *en face* cryosections at a skin depths of 100, 500 and 1500 µm (±20 µm) and stored at −80 °C. Illustrating cutaneous cisplatin biodistribution, drug deposition in depth-specific cryosections was analyzed using ICP-MS. Receiver fluid was collected for ICP-MS quantification of cisplatin permeation through skin.

### Drug quantification: inductively coupled plasma-mass spectrometry (ICP-MS)

Prior to ICP-MS, individual cryosections were transferred to glass vials and dissolved in distilled 65% HNO_3_ for ≥6 hrs at room temperature, followed by 30% H_2_O_2_ overnight. Before analysis, mixtures underwent successive dilution with acid mix (0.65% HNO_3_: 1% HCl). Platinum concentrations (^194^Pt^+^ and ^195^Pt^+^) were measured on an ICP-MS system (ELAN DRC-e or ELAN 6000, PerkinElmer SCIEX, Waltham, MA, USA). Quantification was performed applying an external standard curve, with platinum calibration standards freshly prepared each day (Pt concentration range: 0.1–20 ng/mL, limit of quantification 0.010 ng/mL). All samples were analyzed in duplicate. Measured platinum concentrations (ng/mL) were converted to an absolute amount of cisplatin in each sample (ng), presented as medians with corresponding IQRs. Median cisplatin accumulation in specific skin layers was then calculated based on skin volume (area × thickness; 0.2515 cm^2^ × 0.003 cm) and presented as µg/cm^3^.

For assessment of transdermal cisplatin permeation, receiver fluid was evaporated to dryness (Concentrator Plus, Eppendorf, Hamburg, Germany) and taken into solution with 0.65% HNO_3_: 1% HCl acid mix. Samples were centrifuged at 1,4000 rpm for 10 min (Centrifuge 5430, Eppendorf, Hamburg, Germany), underwent additional acid mix dilution and analyzed using ICP-MS as previously described. Transdermal permeation to receiver compartments is presented as µg/cm^2^, determined by the 0.64 cm^2^ skin surface area over which the drug diffused.

### Drug visualization: laser ablation inductively coupled plasma-mass spectrometry (LA-ICP-MS)

**Figure 4.**
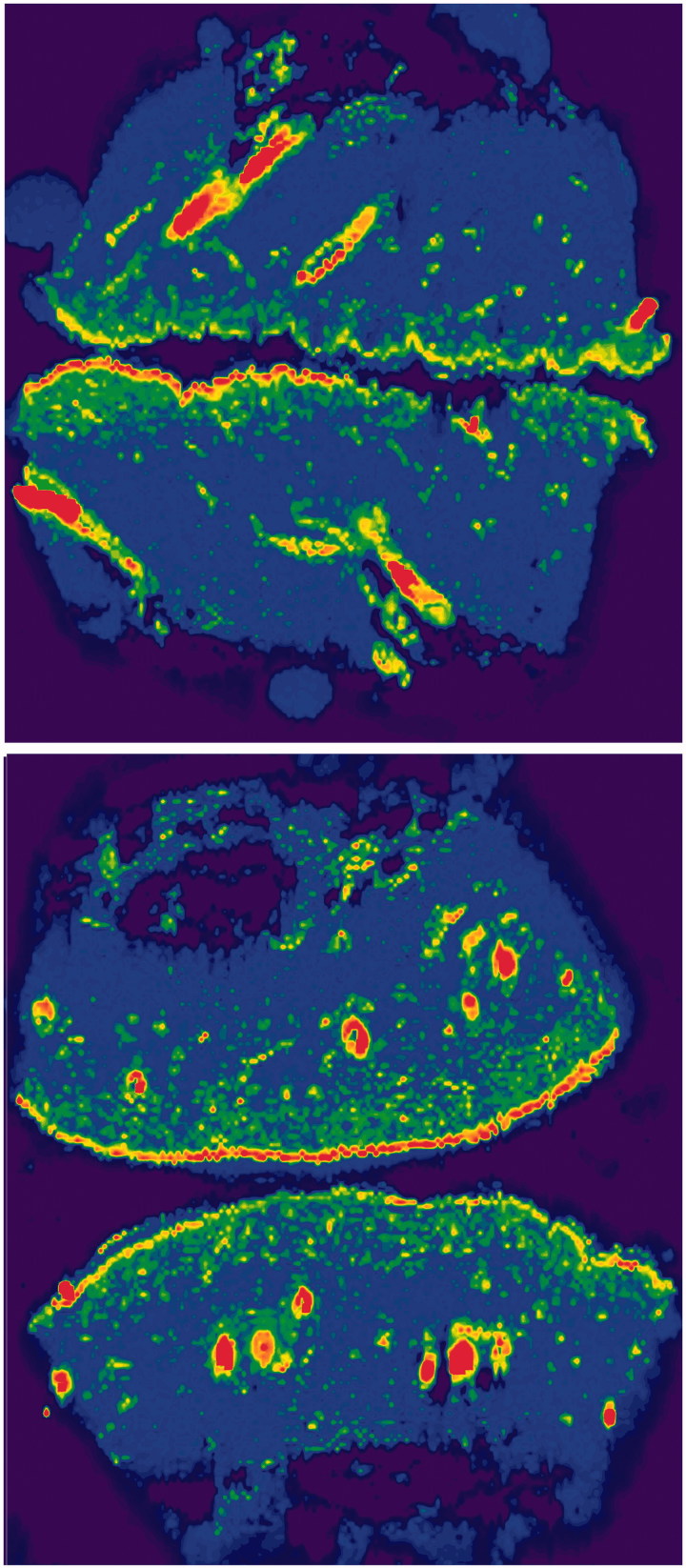
LA-ICP-MS bioimaging of endogenous sulfur (^34^S) in MAZ-DD (above) and non-laser exposed (below) porcine skin, normalized against ^13^C. Notable ^34^S detection is seen corresponding to hair shafts, the outermost stratum corneum and to a lesser extent, the underlying epidermal layers.

Semi-quantitative visualization of cisplatin biodistribution was performed in full-thickness MAZ-DD and non-laser exposed skin samples after 0.5, 4 and 24 h topical drug exposure (Figure 3). LA-ICP-MS analyses were performed using a nanosecond LA unit (NWR193, New Wave Research, Fremont, CA, USA) equipped with an ArF excimer laser source operating at 193 nm as previously described (Persson et al., 2016). Sample transfer from the ablation chamber to the ICP-MS was improved with a Dual Concentric Injector (DCI, New Wave Research, Fremont, CA, USA). The following settings were used: energy: 0.5–0.6 J cm^−2^ (∼20% of maximum energy), scan speed: 272 µm s^−1^, repetition rate: 40 Hz and spot size: 50 µm. All elemental signals were obtained with an Agilent 7900 ICP-MS (Agilent technologies, Manchester, UK), operated in H_2_-mode (3 mL min^−1^). Sample cone depth in the ICP-MS was 3.9 mm, with carrier gas set to 0.89 mL min^−1^. Analyzed isotopes were: ^13^C, ^39^K, ^56^Fe, ^34^S and ^195^Pt (Figures 3 and 4), using integration times of 0.1 (^13^C), 0.03 (^34^S), 0.02 (^195^Pt) and 0.01 (^39^K and ^56^Fe) seconds. The scan cycle was 0.184 seconds. All data was processed with SigmaPlot version 13 (Systat Software Inc., London, UK) and normalized against the samples’ endogenous ^13^C. For a relative distribution analysis of the data of each sample, an additional internal normalization was used where the highest data point in each sample was recalculated and set to index 100. Remaining data points were then multiplied with the same correction factor to produce images where relative, internal skin distribution is displayed (Figure 3).

### Statistics

Descriptive statistics are presented as medians with corresponding IQRs. Non-parametric statistics compared cisplatin deposition in unpaired samples using Kruskal–Wallis and Mann–Whitney *U* tests. For comparison of cisplatin deposition in layers of the same sample, paired Friedman and Wilcoxon tests were applied. *p*-values were exact, 2-sided, and deemed statistically significant if <.05 after Bonferroni correction for multiple comparisons. To estimate the relation between time and AFL-assisted cisplatin uptake in the 1500 µm skin compartment, linear, logarithmic, inverse, power, sigmoid, and exponential mathematical models were fitted to the collected data, revealing power model regression to provide the best fit (Figure 1). Statistical and graphical analyses were performed using SPSS version 24 (IBM Corporation, Arnmonk, NY, USA) and GraphPad Prism version 7.00 (GraphPad Software Inc., San Diego, CA, USA), respectively.

## Results

### Cutaneous cisplatin deposition: quantitative analysis

In non-laser-exposed skin, drug uptake was gradual and limited, with maximum uptake measured in all skin layers at 24 h (Table 1, Figure 1). Cisplatin deposition was primarily confined to the superficial 100 µm skin compartment, with concentrations remaining <2 µg/cm^3^ in the deep 1500 µm layer throughout 0–24 hrs. Cutaneous drug biodistribution was significantly altered by AFL pretreatment, observed consistently in all skin layers. Thus, in contrast to intact skin, concentrations in AFL-exposed samples were generally similar at 100 and 500 µm depth. Deeper deposition at 1500 µm ranged from 41–44% (MAZ-MD) and 32–82% (MAZ-DD) of the examined superficial layers (Table 1).

AFL greatly accelerated cutaneous cisplatin uptake. By 30 minutes, AFL-assisted deposition in deep skin layers was 22-fold greater than in intact skin that had undergone topical cisplatin exposure for a full 24 hours (MAZ-MD vs. untreated: 37.8 µg/cm^3^ vs 1.73 µg/cm^3^ (1500 µm), *p* < .001). Increases were also noted in the superficial compartments despite the short, 30-min exposure time (MAZ-MD vs. untreated (100 µm): 91.5 µg/cm^3^ (30 min) vs 73.8 µg/cm^3^ (24 h) *p* = .04).

Impact of AFL delivery was both time- and skin depth-dependent, with greatest relative enhancement in the deepest skin layer and at later time points (Table 1, Figure 1). At 4 h diffusion time, maximum cisplatin concentrations were 568, 524, and 268 µg/cm^3^, reflecting a 15-, 107-, and 208-fold enhancement versus intact skin at 100, 500, and 1500 µm depth respectively (MAZ-DD vs untreated, *p* < .012) (Figure 2). By 24 hours, a 552-fold increase in maximum cisplatin concentration was noted in the 1500 µm layer (MAZ-DD vs untreated: 954 µg/cm^3^ vs 1.73 µg/cm^3^ (1500 µm), *p* < .001), as compared to lesser 25- and 101-fold enhancements in more superficial 100 µm and 500 µm compartments (MAZ-DD vs untreated: 1874 µg/cm^3^ vs 73.8 µg/cm^3^ (100 µm) *p* < .001; 1713 µg/cm^3^ vs 16.9 µg/cm^3^ (500 µm), *p* < .001).

Increasing median laser channel depth from 620 µm (MAZ-MD) to 912 µm (MAZ-DD) failed to provide statistically significant enhancement in cisplatin delivery at any examined skin layer from 0 to 24 h (Table 1, Figure 1). A trend towards increased uptake was observed at 4 h diffusion time, where deeper laser channels provided greater cisplatin deposition in all skin compartments, most pronounced in the 1500 µm skin layer (MAZ-MD vs MAZ-DD: 449 µg/cm^3^ vs 568 µg/cm^3^ (100 µm), *p* = .084) (Figure 2). By 24 h, deposition in 100 and 500 µm compartments were comparable regardless of laser channel depth (MAZ-MD vs MAZ-DD: 1829 µg/cm^3^ vs 1874 µg/cm^3^ (100 µm), *p* = .981). By contrast in 1500 µm skin layer, a tendency towards greater uptake using deeper laser channels remained (MAZ-MD vs MAZ-DD: 773 µg/cm^3^ vs 954 µg/cm^3^, *p* = .098).

### Cutaneous cisplatin deposition: bioimaging analysis

Quantitative measurements of Pt distribution by ICP-MS was supported by LA-ICP-MS bioimaging, which revealed strikingly improved intracutaneous cisplatin delivery after AFL (Figure 3). Within 30 min, cisplatin was visualized to a skin depth of approx. 1750 µm, with maximum drug detection corresponding to superficial layers and CZs. In the ∼520 µm separating spaces between laser channels, a uniform lateral drug distribution was observed within each skin layer. Quantity and depth of drug detection increased progressively with longer topical exposure from 0.5 to 24 h. Reaching a maximum at 24-h, substantial AFL-assisted cisplatin uptake was seen extending to the deepest layers of skin samples (approx. 3750 µm). Contrastingly in unexposed skin, 24-h topical exposure resulted in minimal drug penetration over outermost skin compartments, with cisplatin primarily concentrated on the cutaneous surface.

### Transdermal cisplatin permeation

AFL led to prominent increases in cisplatin permeation (Table 1, Figure 1). For both laser channel depths, transdermal cisplatin was relatively similar at 30 min, demonstrating 74- and 90-fold increases compared to unexposed skin (untreated vs MAZ-MD: 0.001 µg/cm^2^ vs 0.041 µg/cm^2^*p* = .02; vs MAZ-DD: 0.05 µg/cm^2^*p* = .015). By 4 h, the degree of enhancement using MAZ-MD and -DD had climbed to 2785- and 3192-fold respectively, consistent with rapid drug transit through laser-treated samples. At 24 h, permeation depended significantly on laser channel depth (MAZ-MD vs MAZ-DD: 4.74 µg/cm^2^ vs 11.3 µg/cm^2^, *p* = .001), resulting in 370-, and 884-fold cisplatin using MAZ-MD and MAZ-DD, respectively.

## Discussion

A major and unsolved limitation of topical skin cancer treatment, regardless of the agent applied, is inadequate cutaneous drug delivery (Taveira & Lopez, 2011). Aiming to address this issue, this study showed that AFL provides enhanced and accelerated cisplatin uptake into full-thickness skin. By just 30 min, AFL-assisted drug deposition was significantly greater than the maximal 24-h uptake in non-laser-exposed samples, confirmed by highly sensitive ICP-MS quantification and -bioimaging techniques. Crucially, the greatest impact on cisplatin uptake was observed in the deepest skin layers, where striking enhancements of over 500-fold were noted at 1500 µm depth. Delivery by MAZ-MD and MAZ-DD was not significantly different, although a tendency to greater deep drug deposition using MAZ-DD was observed over time. AFL treatment of skin is a rapid, practical, well-tolerated, as well as available clinical procedure. Our results suggest that AFL-assisted cisplatin delivery is a promising strategy in the pursuit of a more effective topical treatment for NMSC.

Reports of intravenous cisplatin-based chemotherapies in the management of unresectable NMSC predate the 1980s (Salem et al., 1978, Guthrie et al., 1985). Today, despite the advent of more targeted treatments particularly for basal cell carcinoma (BCC), cisplatin remains among the most effective agents against advanced stages of the disease. As such, complete response rates of 37% and 36–57% are reported after systemic therapy for BCC and squamous cell carcinoma (SCC), respectively (Moeholt et al., 1996, Bejar & Maubec, 2014). More recently, local cisplatin treatments have been investigated in clinical and animal studies (Chang et al., 1993, Kitchell et al., 1995, Sersa et al., 1998, Bacro et al., 2000, Hewes & Sullins, 2006, Gupta et al., 2014). Treating two patients with electrochemotherapy with intratumoral injection, Sersa et al. (1998) reported complete responses in 2/2 SCCs and 4/4 BCCs, 8–11 months post-treatment. Using a route more closely resembling AFL-assisted delivery, two studies assessed topical cisplatin iontophoresis for advanced BCC and SCC, observing 5/16 complete- and 7/16 partial responses with best effect in BCCs, small tumors, and after co-administration of epinephrine (Chang et al., 1993, Bacro et al., 2000). These reports support the rationale for developing an improved topical strategy that harnesses cisplatin’s activity against NMSC.

Threshold cisplatin concentrations for adequate NMSC cell inhibition have yet to be described *in vivo*. In the *in vitro* setting, however, a range of inhibitory concentrations are reported for NMSC cells (Featherstone et al., 1991, Uddin et al., 2015, Gil et al., 2016, Olesen et al., 2017). Examined in immortalized keratinocytes (HaCaT) and multiple BCC and SCC lines, Olesen et al. (2017) noted 24-h IC_50_ values of 1.8 µM, 3.5–4.6 µM, and 2.0–2.5 µM respectively, while Featherstone et al. (1991) correspondingly observed 76%–96% reductions in keratinocyte viability following 24-h cisplatin exposure at 4 and 8 µM. Measured at 1500 µm skin depth within 30 min of drug application, we observed cisplatin skin concentrations of >100 µM. In the clinical setting, intratumoral cisplatin concentrations are typically reported in the range of ∼0.5–4.5 µg/g tissue 0–72 hours after systemic infusion for head and neck SCC, assuming a skin density of 1.1 g/cm^3^ (, Hecquet et al., 1985, Hecquet et al., 1987, Gouyette et al., 1986, Holding et al., 1991). In this study, greater concentrations of >89.8 and 29.5 µg/g skin were calculated in 500 and 1500 µm skin layers within 30 min. Though optimal concentrations of topical cisplatin have yet to be determined *in vivo*, relative to both preclinical and clinical literature, our findings underscore the feasibility of achieving high therapeutic levels of drug throughout skin using AFL.

For hydrophilic drugs, impact of LADD has been shown to depend on laser channel density and depth. Increasing MAZ depth is reported to enhance topical delivery of a range of compounds, including anticancer agents (Wenande et al., 2017a). This depth-dependence is theorized to be a reflection of improved hydrophilic drug partitioning from (1) vehicle into the more hydrophilic medium occupying channels shortly after irradiation and (2) from the medium into the surrounding skin tissue (Erlendsson et al., 2016). While increased transdermal cisplatin permeation and a tendency towards greater uptake in the deepest skin layer using MAZ-DD was observed in this study, differences in cutaneous deposition between MAZ-MD and MAZ-DD did not reach significance despite the drug’s negative Log *P.* A potential explanation may be cisplatin’s sparing water-solubility (0.253 g/100 ml), which could limit the drug’s ability to take advantage of deeper channels (O'Neil, 2006). Our data also reveals uniform dermal drug distribution in the ∼520 µm lateral spaces between laser channels, and cisplatin diffusion from MAZs may saturate in the absence of a strong drug concentration gradient. It follows, that increasing laser channel depth beyond a certain threshold may not necessarily provide further enhancement in drug delivery. Indicating the possibility of a non-linear relationship between MAZ depth and drug uptake, we recently showed that increasing MAZ depth from 104, 322, to 565 µm did not lead to comparable degrees of enhancement in 5-FU deposition in *ex vivo* skin (Wenande et al., 2017b). The current study's results support this theory, as differences in skin uptake using 620 vs. 912 µm channel depths were even lower. Whether the same relationship between channel depth and drug uptake exists under *in vivo* conditions remains unexamined.

Early toxicological studies describe cisplatin as a mild skin irritant following 24-h topical exposure (INCHEM, 1991). Using the same 1 mg/mL cisplatin solution as applied here, normal appearances of *in vivo* porcine and murine skin are reported after simple topical application for 1–12 h (Miatmoko et al., 2015, Wenande et al., 2018). Delivered by AFL however, the agent’s safety profile differs due enhanced skin uptake. We previously assessed this in healthy skin of live pigs. After 60 mins topical AFL-assisted cisplatin, increased erythema scores were observed over 5 days versus AFL alone. Meanwhile, cisplatin remained undetectable in plasma measured from 0–120 hours, despite >100 cm^2^ skin exposures (Wenande et al., 2018). Though additional studies are needed, risks of AFL-assisted cisplatin therapy thus far appear acceptable.

The study has limitations. While illustrative and widely accepted, data derived from *ex vivo* porcine skin and an infinite dose model cannot be directly extrapolated to the clinical setting. ICP-MS relies on detection of Pt rather than the intact cisplatin molecule, thereby providing an assessment of total skin deposition as opposed to activated or DNA-bound intracellular species. Though high chloride concentrations in the cisplatin solution and cutaneous extracellular environment theoretically reduce ‘off target’ binding of cisplatin prior to entering cells, the presence of nucleophilic elements in skin structures might result in extracellular retention of cisplatin. For example, sulfur groups associated with keratin or proteoglycans in skin, could potentially bind the drug after uptake. However, we found no correlation between cutaneous distribution of cisplatin and sulfur-rich skin structures (Figure 4). However, Chang et al. (2016) recently detected extensive cisplatin binding to dermal collagen four hours post intraperitoneal administration, after which the drug was slowly released over 7 days (Chang et al., 2016). Binding and subsequent release of cisplatin from the dermal extracellular matrix may actually be an advantage, due to sustained dermal drug availability to cells. Past studies interestingly indicate that up to 10% of the activity of released platinum species is retained (Brouwers et al., 2008). At present, further investigation is needed to examine whether cutaneous cisplatin retention results in reduced treatment efficacy or leads to gradual release of the drug to adjacent NMSC cells over time.

In conclusion, illustrated by quantitative and semi-quantitative bioimaging analyses, this study demonstrates that AFL provides rapid, greatly increased and uniform delivery of the anticancer agent cisplatin deep into full thickness skin. Improved cutaneous uptake of this potent chemotherapeutic may prove useful in the development of a new, local and minimally-invasive treatment for dermatological neoplasms.
